# A review of the surveillance systems of influenza in selected countries in the tropical region

**DOI:** 10.11604/pamj.2014.19.121.4280

**Published:** 2014-10-01

**Authors:** Melvin Sanicas, Eduardo Forleo, Gianni Pozzi, Doudou Diop

**Affiliations:** 1 Faculty of Medicine and Surgery, University Degli Studi of Siena, Siena Italia; 2 Novartis Vaccines & Diagnostics, Siena, Italy

**Keywords:** Tropical region, influenza surveillance, epidemics of respiratory

## Abstract

Influenza viruses cause annual epidemics of respiratory tract disease that affect all age groups. Many developing countries do not have an influenza surveillance system or adequate laboratory capacity for virus detection. The objective of this study was to describe the influenza surveillance systems in the different countries in the tropics and to identify outstanding research needs. A questionnaire was designed and sent to 52 NICs and MoHs in the different countries in tropical Asia and Africa to gather information on the surveillance systems, sentinel sites, specimen and data collection, and laboratory testing. Replies were received from 32 NICs and MoHs (61.5% response) – 17 were located in tropical Asia and 15 in Africa. There are 20 WHO recognized NICs in tropical Asia and 14 in tropical Africa, all with virus isolation and polymerase chain reaction (PCR) testing capacity. Of the Asian countries, only Hong Kong and Singapore reported that the patient population from the sites represents the broader community. In tropical Africa, only Senegal has sentinel sites distributed all over the country contributing to the geographic representativeness of the surveillance system. The rest of the countries in Africa have just established their influenza surveillance system in the past decade and are working toward geographic expansion of the ILI and SARI sites. Limited laboratory capacity or infrastructure to perform influenza surveillance makes difficult to justify the importance of influenza vaccine or other influenza control measures as a strategy for improving population health in the tropical region.

## Introduction

Influenza is a highly communicable acute respiratory disease which affects populations for centuries [1]. The influenza virus can cause illness in individuals of all ages, results in repeated infections throughout life, and is responsible for annual worldwide epidemics of varying severity. Every year, influenza causes several hundred million cases of which about three to five million of severe illnesses [2–4] and an estimated 500,000 to 1 million deaths [5, 6]. The high genetic mutability of the virus requires continuous monitoring of influenza cases during each influenza season by the WHO and national surveillance systems in order to identify the prevalent virus strains in the population. In recent years, concerted efforts from the WHO Member States, through training workshops, acquisition of equipments and reagents, have led to significant increases in trained personnel and equipped laboratories leading to expansion in both geographical surveillance coverage and in the capacities of influenza laboratories. These efforts have been supported by a broad range of national and international agencies. Moreover, the 2009 H1N1 pandemic provided an opportunity to gain experience, learn important lessons on influenza surveillance and pandemic planning. The pandemic increased awareness of the threat posed by influenza and led to a renewed enthusiasm towards capacity-building for influenza laboratories and the need to set up influenza laboratories in countries where there are none [7–10]. Many developing countries do not have an influenza surveillance system or adequate laboratory capacity for virus detection. This project also aims to describe the existing influenza surveillance systems in the different countries in the high respiratory-endemic disease regions of tropical Asia and Africa to identify outstanding research needs.

## Methods

To understand the different influenza surveillance systems in the different countries in the tropical region, a questionnaire was designed to gather information specifically on the surveillance system, sentinel sites, specimen and data collection, and laboratory testing. The questionnaire was sent to the different National Influenza Centers and/or Ministry of Health. Additional information was obtained either online from the Ministry of Health websites of the different countries. Annual reports and presentations made by the National Influenza Centers or Ministries of Health to International Conferences (e.g. Annual African Network for Influenza Surveillance and Epidemiology) were also included.

## Current status of knowledge

Questionnaires were sent to 52 National Influenza Centers (NICs) and Ministries of Health (MOHs) in the different countries in Tropical Asia and Africa. Replies were received from 32 NICs and MOHs (61.5% response). In this study 32 countries were included among which 17 were located in tropical Asia. For Africa, only 15 countries were included. These were the countries whose National Influenza Centers responded to the questionnaires sent or responded by providing presentations or publications from the country.

### Surveillance Systems in Tropical Asia

In Asia, the Tropic of Cancer runs from west to east through Saudi Arabia, United Arab Emirates, Oman, India, Myanmar, Southern China and Taiwan and the Tropic of Capricorn runs through Australia. Some 19 countries including 4 in the Middle East (Yemen, parts of Saudi Arabia, Oman, and United Arab Emirates) are situated in the tropical region. India, in southern Asia, lies mostly in the tropics, and all countries of Southeast Asia are tropical countries. Places located in the Pacific including parts of Australia, Micronesia, the Marshall Islands, Kiribati, and most of the other island nations of Oceania in the South Pacific are also considered tropical.

In tropical Asia, Singapore and Indonesia were the first countries to conduct influenza surveillance in the 70s. New Caledonia, Hong Kong, and Taiwan followed in the 1990s. All the rest started after 2000 with Laos, Bangladesh, and Sri Lanka starting in 2007. ILI and SARI surveillance have been initiated in all the countries in tropical Asia except for Malaysia and Cambodia. Malaysia currently focuses on ILI while Cambodia does ILI and ALRI (acute lower respiratory infection) surveillance. All countries publish their influenza surveillance data in WHO FluNet except for Saudi Arabia and Taiwan (Table 1, Table 2).

**Table 1:** Summary of influenza surveillance in tropical Asia

Country	Date started	sites	ILI/SARI	Able to mesure burden of disease	Data Published	Submitted to WHO FluID/FluNet
**Middle East**						
**Saudi Arabia**	2004	6 ILI and SARI sites; 2 temporary Hajj sites	Both	**X**	**X**	**X**
**Oman**	2001	14 ILI and SARI sites	Both	**X**	**√**	**√**
**Northern Asia**						
**Hong Kong**	Early 90s	64 public out-patient clinics, 40 private practice clinics for ILI; all hospitals for SARI	Both	**√**	**√**	**√**
**Taiwan**	1999	12 sites; hospitals participating in Real-Time Outbreak & disease Surveillance	Both	**√**	**√**	**X**
**Myanmar**	2005	7 ILI and SARI sites, only in major cities	Both	**√**	**X**	**√**
**Laos**	2007	3 hospitals in & around the capital city	Both	**X**	**X**	**√**
**Thailand**	2004	13 Medical Centers all over the country, ILI in >900 hospitals & health centers	Both	**√**	**√**	**√**
**Vietnam**	2005	4 central referral hospitals, 2 provincial hospitals, 7 district hospitals, 2 urban polyclinics	Both	**X**	**X**	**√**
**Southern Asia**						
**Bangladesh**	2007	6 government, 6 private medical hospitals; 14 district hospitals	Both	**√**	**√**	**√**
**India**	2004	7 centers for ILI & SARI	Both	**√**	**X**	**√**
**Sri Lanka**	2007	20 ILI sites, 3 SARI sites	Both	**X**	**X**	**√**
**South East Asia & South Pacific**						
**Cambodia**	2006	2 hospitals for SARI, 5 out-patient sites for ILI	ALRI, ILI	**X**	**X**	**√**
**Philippines**	2005	34 sites for ILI; 5 sites in 1 city for SARI	Both	**√**	**√**	**√**
**Malaysia**	2003	16 sentinel private clinics in the central part of the capital	ILI	**X**	**√**	**√**
**Singapore**	1973	98 private clinics, 18 public clinics for ILI, all hospitals for SARI	Both	**√**	**√**	**√**
**Indonesia**	1975	44 sentinel sites	Both	**X**	**√**	**√**
**New Caledonia**	1998	9 sentinel sites	Both	**√**	**√**	**√**

**X** = no **√** = yes

**Table 2:** Influenza surveillance collaborations in tropical Asia

**Country**	Collaboration
**Saudi Arabia**	WHO, NAMRU-3
**Oman**	WHO
**Hong Kong**	WHO, University of Hong Kong
**Taiwan**	WHO, CDC
**Myanmar**	WHO, Nigata University
**Laos**	WHO, CDC, Thai MoPH, NAMRU-2
**Thailand**	WHO, CDC
**Vietnam**	WHO, CDC, Institut Pasteur
**Bangladesh**	WHO, CDC
**India**	WHO, CDC
**Sri Lanka**	WHO, CDC, University of Hong Kong
**Cambodia**	WHO, CDC, IP, Thai MoPH, Laos MoH, NAMRU-2
**The Philippines**	WHO, CDC
**Malaysia**	WHO
**Singapore**	WHO, CDC, NAMRU-2
**Indonesia**	WHO, NAMRU-2
**New Caledonia**	WHO, Institut Pasteur

**CDC**: United States Center for Disease Control and Prevention, **MoH**: Ministry of Health, **MoPH**: Ministry of Public Health, **NAMRU**: Naval Medical Research Unit, **WHO**: World Health Organization

Source: WHO April 2012, NAMRU-2, US CDC

### Surveillance Systems in Tropical Africa

In Africa, the Tropic of Cancer runs from west to east through the African countries Western Sahara, Mauritania, Mali, Algeria, Niger, Libya, Chad, and Egypt. The Tropic of Capricorn runs from west to east through the African nations Namibia, Botswana, South Africa and Mozambique. The only countries that cannot be called tropical are Morocco and Tunisia in the north and Lesotho, South Africa, and Swaziland in the South. The rest lie entirely or partly within the tropics.

In West Africa, from only 2 countries conducting influenza surveillance in 2006, 6 additional countries have established a functional in-country surveillance system. Mauritania has just started Flu Surveillance. From only 1 functional WHO recognized National Influenza Centers in 1996 (Senegal), there are now 4 NICs in the region (Senegal, Nigeria, Ghana, Cote d'Ivoire). In Middle Africa only Cameroon, Angola, Central African Republic, and the Democratic Republic of Congo have functioning Influenza Surveillance systems. Chad, Equatorial Guinea, Gabon, and Sao Tome and Principe currently do not have Influenza Surveillance systems. In East Africa, the countries with Influenza Surveillance systems include Ethiopia, Tanzania, Kenya, Uganda, and Rwanda. All these countries have National Influenza Centers recognized by the WHO. In the Horn of Africa, only Ethiopia has a functioning Influenza Surveillance system. In Southern Africa, Madagascar, Mozambique, Zambia, and South Africa have Influenza Surveillance systems. There are 3 WHO-recognized National Influenza Centers in Southern Africa – 1 in Madagascar, and 2 in South Africa. South Africa is not included here as most of country is located outside of the tropics (Table 3, Table 4).

**Table 3:** Summary of influenza surveillance in tropical Africa

Country	Date started	Sites	ILI/SARI	Able to measure burden of disease	Data Published	Submitted to WHO FluID/FluNet
**West Africa**						
**Cote d'Ivoire**	2008	16 ILI sites, 8 SARI sites	Both	**X**	**√**	**√**
**Senegal**	1996	6 hospitals in the capital city, 24 sentinel sites in different regions	ILI	X	**√**	**√**
**Mauritania**	2010	1 ILI site, 1 SARI site	Both	**X**	**---**	**---**
**Ghana**	2007	15 ILI sites, 3 SARI sites	Both	**X**	**X**	**√**
**Togo**	2009	1 ILI site, 1 SARI site	Both	**X**	**X**	**√**
**Mali**	2009	4 sentinel sites in different parts of the capital city	Both	**X**	**X**	**√**
**Burkina Faso**	2009	1 ILI site, 1 SARI site	Both	**X**	**X**	**√**
**Sierra Leone**	2011	4 sentinel sites in different parts of the capital city	Both	**X**	**X**	**√**
**Niger**	2009	12 ILI sites, 12 SARI sites	Both	**X**	**---**	---
**Nigeria**	2008	4 ILI sites and 4 SARI sites scattered across 4 of the 6 geopolitical zones	Both	**X**	**X**	**√**
**Middle Africa**						
**Cameroon**	2010	37 clinics, hospitals, and infirmaries in 3 regions of the country	ILI	**X**	**X**	**√**
**DR Congo**	---	5 sites in Kinshasa, 2 sites in Bas Congo	Both	**X**	**X**	**√**
**Angola**	2003	2 SARI sites, 2 ILI sites	Both	**X**	**X**	**√**
**East Africa**						
**Ethiopia**	2008	2 regional hospitals for SARI, 3 primary healthcare centers for ILI	Both	**X**	**X**	**√**
**Kenya**	2006	11 SARI sites, 27 ILI sites	Both	**X**	**X**	**√**
**Rwanda**	2008	6 SARI sites, 6 ILI sites	Both	**X**	**X**	**√**
**Tanzania**	2009	6 SARI sites, 6 ILI sites	Both	**X**	**X**	**√**
**Uganda**	2007	13 sentinel sites in the 4 regions of the country	Both	**X**	**X**	**√**
**Southern Africa**						
**Madagascar**	2007	ILI: 34 clinical sites, 9 sites for biological surveillance; SARI: 17 clinical sites, 2 sites for biological surveillance	Both	√	√	√
**Zambia**	2008	2 out-patient clinics for ILI, 2 hospitals for SARI	Both	**X**	**X**	√

**X** = no **√** = yes

**Table 4:** Influenza surveillance collaborations in tropical Africa

**Country**	Collaboration
**Cote d'Ivoire**	WHO, CDC, Institut Pasteur
**Senegal**	WHO, Institut Pasteur, AMP (SISA), Mauritana, Guinea, Cape Verde
**Ghana**	WHO, NAMRU-3
**Mali**	WHO, NAMRU-3, UM
**Burkina Faso**	WHO, NAMRU-3
**Togo**	WHO, NAMRU-3
**Sierra Leone**	WHO, IP, AMP (SISA)
**Cameroon**	WHO, AMP (SISA)
**Niger**	WHO, Institut Pasteur
**Nigeria**	WHO, CDC, AMP (SISA)
**Central African Rep**	WHO, Institut Pasteur
**Sudan**	WHO, University of Khartoum
**DR Congo**	WHO, CDC
**Angola**	WHO, CDC
**Ethiopia**	WHO, CDC
**Kenya**	WHO, CDC, AFHSC-GEIS
**Rwanda**	WHO, CDC, AMP (SISA)
**Tanzania**	WHO, CDC, AFHSC-GEIS
**Uganda**	WHO, CDC, MUWRP
**Madagascar**	WHO, Institut Pasteur, CDC, FRA MoH, SEGA, SP
**Zambia**	WHO, CDC, AMP (SISA)
**Mozambique**	WHO, CDC

**AMP (SISA)**: Agence de Medecine Preventive (Strengthening Influenza Sentinel Surveillance in Africa), **AFHSC-GEIS**: Armed Forces Health Surveillance Center-Global Emerging Infections Surveillance and Response System, **CDC**: United States Center for Disease Control and Prevention, **FRA MoH**: Ministry of Health, France, **MUWRP**: Makerere University Walter Reed Program, **NAMRU-3**: Naval Medical Research Unit 3, **SEGA**: la surveillance epidemiologique dans l'ocean Indien, **SP**: Sanofi Pasteur, **UM:** University of Maryland, **WHO**: World Health Organization Source: WHO April 2012, US CDC 2010, ANISE 2012

There are 21 WHO-recognized National Influenza Centers in Tropical Asia: 3 in India, 2 in Vietnam, 2 in Malaysia, and 1 each in Bangladesh, Indonesia, Myanmar, Sri Lanka, Thailand, Cambodia, Hong Kong SAR, Taiwan, Fiji, Papua New Guinea, New Caledonia, Laos, The Philippines, and Singapore. Except for Maldives, Brunei, and East Timor, all of the countries in the Tropical region in Asia have Influenza Surveillance systems. There are 14 WHO-recognized National Influenza Centres in Tropical Africa: Cote d'Ivoire, Senegal, Ghana, Nigeria, Cameroon, DR Congo, Angola, Uganda, Rwanda, Sudan, Ethiopia, Kenta, Tanzania, and Madagascar.

### Influenza Surveillance Case Definitions

Human Influenza surveillance comprises of 2 components: Influenza-Like Surveillance (ILI) and Severe Acute Respiratory Infection (SARI). ILI and SARI surveillance have been initiated in all the countries in tropical Asia except for Malaysia and Cambodia. Malaysia currently focuses on ILI while Cambodia does ILI and ALRI (acute lower respiratory infection) surveillance. ILI and SARI surveillance have been initiated in all the countries in tropical Africa except for Cameroon and Senegal which only focus on ILI surveillance for the moment. The surveillance systems in tropical Asia and Africa use the existing World Health Organization (WHO) case definition for Influenza-like illness [11] and incorporate the WHO guidance to define severe acute respiratory infection in adults and children [12–14].

### Sites, Laboratory, and Infrastructure

There are 20 WHO recognized NICs in tropical Asia and 14 in tropical Africa. All with virus isolation and polymerase chain reaction (PCR) testing capacity. Previously the methods employed for virus detection were mainly virus isolation and the haemagglutination inhibition test. Subsets of the specimens undergo viral culture and characterization. The on-going presence of the highly pathogenic avian A (H5N1) influenza virus since early 2004 increased the need for PCR (polymerase chain reaction) as part of the diagnosis process in order to improve the accuracy and efficiency of testing. Virologists and laboratory technicians in the NIC of the countries have been trained overseas on influenza laboratory diagnosis and detection. For most of the NICs, epidemiologists are full-time employees. Relevant technical personnel have been trained by staff from the WHO or US CDC or Institut Pasteur.

In terms of surveillance sites, for Asia, only Hong Kong and Singapore reported that the patient population from the sites represents the broader community. This is most probably due to the size of the state and the healthcare IT network and integrated surveillance system. For India, samples are representing almost all the regions in the country, but not the individual states. For archipelagos like The Philippines and Indonesia with more than 7,000 and 13,000 islands respectively, obtaining a truly representative sample may be a challenge. However, the majority of the population is concentrated on the major cities, i.e. Metro Manila and Java where several sentinel sites are located. The rest of the countries in Asia are working toward geographic expansion of the ILI and SARI sites. Maldives, Brunei and East Timor do not have a National Influenza Center.

In tropical Africa, only Senegal has sentinel sites distributed all over the country contributing to the geographical representativeness of the surveillance system. The rest of the countries in Africa have just established their influenza surveillance system in the last decade and are working towards geographic expansion of the ILI and SARI sites. Several countries do not have the laboratory capacity or infrastructure to perform influenza surveillance. These countries include the following: Benin, Burundi, Cape Verde, Chad, Comoros, Djibouti, Equatorial Guinea, Eritrea, Gabon, Guinea, Guinea Bissau, Lesotho, Liberia, Malawi, Namibia, Botswana, Mauritania, Mauritius, Sao Tome and Principe, South Sudan, Seychelles, Somalia and Zimbabwe.

The burden of influenza in the tropical Asia and Africa is still not fully understood. However there have been some studies to learn more, particularly in Thailand, Myanmar, and India. Philippines and Bangladesh are currently conducting burden of disease studies. Defining the epidemiology and burden of influenza is high on the priority list of the WHO Southeast Asian Region and Western-Pacific region 2011 Bi-regional Plan for Further Strengthening National Influenza Surveillance [15]. In Africa, Senegal is currently conducting a vaccine effectiveness study (Clinicaltrials.gov NCT00893906) which will help to elucidate burden of seasonal influenza [16].

### Specimen collection, epidemiological capacity, reporting

Respiratory specimens (either nasal swab or throat swabs) are collected early from ILI and SARI patients using established protocols. For most of the countries in tropical Asia and Africa, it is not feasible to collect specimens from all patients therefore systematic sampling is done. There is no generic number of specimens collected in each site and this depends on several factors like seasonality and attack rates [12, 13]. Most of the countries have their own sample collection form, however, the forms require similar information including: name, date of birth, sex, address, onset of symptoms, date of collection of epidemiological data, clinical signs and symptoms, type of specimen collected and date of collection, pre-existing medical conditions). In most of the NICs, epidemiologists are full-time employees who are in charge of outbreak investigation and response, risk communication and analysis of ILI and SARI data. Relevant technical personnel have been trained by staff from the WHO or US CDC or Institut Pasteur. Analyses of the surveillance data are performed by trained personnel and the analyses include weekly frequencies of SARI cases and laboratory-confirmed influenza cases as well as the portion of tested patients who are influenza virus positive. Surveillance data is submitted to WHO FluNet and most of the national laboratories work with a WHO Collaborating Center laboratory to submit sample virus isolates for vaccine strain selection. The frequency of data collection and reporting vary from country to country. Most countries do a weekly collection of data and a monthly reporting to the respective Health Ministries and the WHO FluNet.

### Partnerships and collaboration

All NICs and MoHs in tropical Asia and Africa collaborate with the WHO. Initiatives for building national capacity in the countries in tropical Asia and Africa include cooperative agreements with the US Center for Disease Control and Prevention (CDC) in Atlanta, United States of America. In tropical Africa countries like Nigeria, DR Congo, Angola, Ethiopia, Kenya, Rwanda, Tanzania, Uganda, Zambia and Mozambique these agreements have been instrumental in the establishment and improvement of national influenza surveillance capabilities. In Ghana, Mali, Burkina Faso, and Togo influenza surveillance receive assistance from the CDC via the US Naval Medical Research Unit-3 (NAMRU-3).

In some countries, the NICs are hosted within the International Network of Pasteur Institutes. In tropical Africa Cote d’ Ivoire, Senegal, Sierra Leone, Central African Republic, and Madagascar are part of the network. In tropical Asia Vietnam, Cambodia, and New Caledonia are part of the Institut Pasteur network. In tropical Asia, Saudi Arabia, Cambodia, Singapore, and Indonesia collaborate with NAMRU. Saudi Arabia collaborates with NAMRU-3 while the other Asian countries work with NAMRU-2.

## Conclusion

There are existing influenza surveillance systems in several countries in Asia and Africa. WHO and different organizations such as the US CDC, NAMRU, and Institute Pasteur lend support to National Influenza Centers and laboratories in Asia and Africa. Surveillance will allow determination of the rates of influenza and a description of the clinical characteristics of the disease. With such epidemiologic data, public health officials will have better data in order to formulate influenza control strategies for either seasonal or pandemic influenza. Most of the countries are working toward geographic expansion of the ILI and SARI sites. Very few countries in tropical Asia and Africa have the capacity to measure the diseases burden of influenza. About half of the countries in Africa still do not have the laboratory capacity or infrastructure to perform influenza surveillance. This makes it difficult to justify the importance of influenza vaccine or other influenza control measures as a strategy for improving population health. Governments have taken steps to identify and focus on gaps in knowledge as indicated by the increasing development of surveillance networks, the organization of scientific congresses such as the African Influenza Scientific Symposium, ANISE meetings and the WHO (AFRO, WPRO, SEARO) meetings. Although these efforts have not yet resulted in a great increase in published manuscripts and data, this situation should change within the next few years. It is important to encourage national laboratories to publish results of their studies. Collaborations between countries should be strengthened for efficient utilization of laboratory services and capacities.
